# Beer Consumption and Health: A Bibliometric Analysis of Five Decades of Scientific Literature

**DOI:** 10.3390/foods15132303

**Published:** 2026-06-27

**Authors:** Ana Garcia-Megias, Diego Diaz-Milanes

**Affiliations:** 1 Loyola Health Policy Institute, Universidad Loyola Andalucía, 41704 Seville, Spain; agarciamegias@al.uloyola.es; 2 Department of Quantitative Methods, Universidad Loyola Andalucía, 41704 Seville, Spain; 3 Health Research Institute, University of Canberra, Canberra, ACT 2617, Australia

**Keywords:** beer consumption, health, drinking patterns, bibliometric analysis, bibliometrix, science mapping

## Abstract

**Background/Objectives**: Beer consumption has attracted increasing scientific attention due to its widespread prevalence and potential implications for public health. This study aims to examine the evolution and characteristics of scientific output on beer consumption using a bibliometric approach. **Methods**: A systematic search was conducted in the Web of Science database covering the period 1974–2024, identifying 2609 relevant documents following a rigorous screening process. **Results**: The findings indicate a sustained increase in publication volume, particularly since 2000, with significant contributions from countries such as the United States and the United Kingdom. The analysis identifies the most influential sources, authors, institutions, and collaboration networks, as well as the principal thematic areas, broadly categorized into clinical–nutritional and socio-epidemiological perspectives. **Conclusions**: This study provides an integrated overview of current research on beer and health and underscores the need for more targeted studies focusing on vulnerable populations and specific contexts.

## 1. Introduction

Beer is one of the most widely consumed beverages worldwide. It is the most consumed alcoholic beverage globally and ranks among the highest in total volume of intake, after water and tea [1]. As a fermented alcoholic beverage produced mainly from water, malted cereals, hops, and yeast, beer contains ethanol as well as non-alcoholic compounds, including polyphenols, minerals, vitamins, and other fermentation-derived substances. These components have contributed to scientific interest in their potential health-related effects [2]. In Spain, beer likewise occupies the leading position among alcoholic beverages across age groups, with consumption increasing progressively up to approximately 44 years of age and then tending to stabilize [3]. Beyond these consumption figures, beer’s public health relevance stems from its unique position as an accessible, relatively inexpensive product that is deeply embedded in everyday lifestyles and social life across diverse cultural settings [4]. As such, beer consumption represents a common and socially patterned exposure that can operate as a determinant of health.

From a public health perspective, alcohol consumption patterns and their associated consequences constitute a critical area of investigation [5]. Drinking behaviors are typically classified into moderate consumption, binge drinking, and habitual excessive consumption [6]. These patterns have substantial implications not only for individual health outcomes but also for broader social and economic systems. Importantly, beer often serves as a primary vehicle through which these patterns are expressed in many populations, given its widespread availability and strong social acceptability in numerous contexts [7].

Understanding beer consumption as a health determinant also requires a clear conceptualization of “health.” According to the World Health Organization (WHO), health is a holistic state of complete physical, mental, and social well-being, rather than merely the absence of disease. Within this framework, the potential implications of beer consumption extend beyond specific clinical endpoints to include mental health, social functioning, and well-being, as well as the contextual factors that shape drinking behaviors. Consequently, it is essential to consider not only the health-related effects of beer consumption but also the broader determinants that influence it, including individual characteristics, social environments, economic conditions, and cultural norms.

Over recent decades, scientific interest in alcohol-related research has expanded considerably [8]. Research groups examined the health effects of beer and other alcoholic beverages, the behavioral and social determinants of drinking patterns, and their relationship with overall health and well-being. The association between alcohol consumption and health outcomes has long been the subject of sustained scientific debate [9]. Historically, a J-shaped relationship has been proposed, whereby low to moderate levels of consumption were associated with certain protective effects—particularly regarding cardiovascular health—when compared with abstention [10]. However, these findings have been increasingly challenged by more recent investigations using more rigorous methodological designs and improved control of confounding factors [11,12], although the issue remains an area of active scientific discussion [13]. In parallel, recent evidence highlights the importance of tailoring alcohol consumption guidelines to individual and contextual characteristics, including age, sex, and geographical setting, and emphasizes the development of targeted preventive strategies for younger populations, given alcohol’s substantial contribution to the global burden of disease [13].

In this context, bibliometric analysis constitutes a robust and widely recognized methodological approach for systematically assessing the structure and evolution of scientific knowledge [14,15]. By quantitatively examining patterns of academic production, bibliometric methods enable the identification of publication trends, influential authors and institutions, collaborative networks, and emerging thematic domains [16]. This approach is especially valuable in research areas characterized by high publication volume and thematic heterogeneity, such as the literature connecting beer consumption with health outcomes and their underlying determinants.

Despite the growing volume of research on alcohol and health, bibliometric work specifically focused on beer consumption is scarce and narrow in scope. To date, the available studies have largely concentrated on beer’s chemical composition and production methods [1], treating beer primarily as a product rather than examining consumption as a determinant of health. However, a beer-specific bibliometric analysis is warranted because beer is not only a widely consumed alcoholic beverage, but also a socially normalized exposure whose study is dispersed across food science, nutrition, epidemiology, behavioral sciences, addiction research, and public health [1,17,18]. This gap limits a systematic understanding of how the evidence base on beer consumption and health has developed, where research efforts have been concentrated, and which themes, populations, and outcomes remain underexplored.

Accordingly, the aim of the present study is to analyze the scientific output on beer consumption in relation to health using a bibliometric approach. Specifically, we seek to identify major research trends, the most influential authors and journals, and the principal institutional and international collaboration networks shaping this field. By providing a structured map of the literature, this study aims to support a clearer understanding of beer’s role as a common and socially embedded determinant of health and to highlight priorities for future research and public health action.

## 2. Materials and Methods

### 2.1. Data Sources and Search Strategy

The data used in this study were retrieved from the Web of Science Core Collection (WoSCC) on 14 May 2025. WoSCC was selected because it provides standardized bibliographic and citation metadata, as well as complete cited references, which are essential for citation-based science mapping (e.g., co-citation and collaboration networks) using Bibliometrix/Biblioshiny. Relying on a single, well-curated source also helps preserve comparability and replicability by minimizing inconsistencies that can arise during multi-database merging and deduplication. The following search strategy was applied: ((TI = (beer) OR AB = (beer)) OR (TI = (“beer consumption”) OR AB = (“beer consumption”)) OR (TI = (“beer intake”) OR AB = (“beer intake”))).

**Figure 1:**
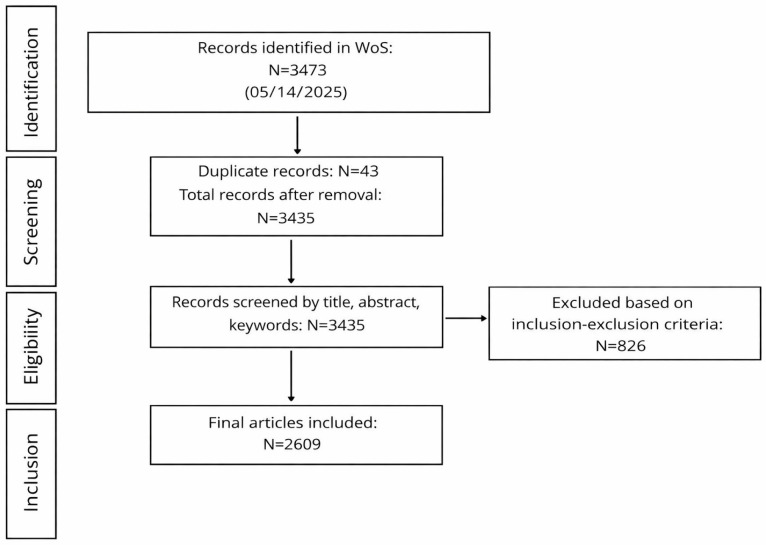
Flow diagram of the study selection process.

To ensure compatibility with bibliometric analysis tools, all records were exported with complete metadata (“Full Record and Cited References”). Following the methodological framework proposed by Aria and Cuccurullo [16], we applied a structured five-stage workflow: (1) research design, (2) data collection, (3) data analysis, (4) visualization, and (5) interpretation. In addition, to improve transparency and replicability in the document selection process, we included a flow diagram summarizing the identification, screening, eligibility assessment, and exclusion of records (Figure 1).

### 2.2. Inclusion and Exclusion Criteria

To ensure thematic consistency and the relevance of the analyzed documents, specific inclusion and exclusion criteria were applied. First, the publication period was limited to the interval between 1974 and 2024 to capture both the historical evolution and current trends related to the phenomenon under study. Additionally, the language of the documents was restricted to English, given its predominance in indexed scientific literature and to minimize potential duplication. Regarding document type, only original research articles, review articles, and data papers were included, while other categories such as conference proceedings, editorials, and letters to the editor were excluded, as they do not directly provide empirical evidence or systematic syntheses of knowledge.

Finally, relevant subject areas were defined to align with the study objectives. The included documents belonged to the fields of public health, psychology, behavioral sciences, psychiatry, general internal medicine, nursing, addiction studies, health services, and nutrition and dietetics. This thematic selection allowed the analysis to focus on research examining beer consumption from a holistic perspective, encompassing health-related outcomes as well as behavioral and social dimensions.

Duplicate records were identified and removed using the Rayyan online platform. Subsequently, two authors independently screened the titles, abstracts, and keywords of the retrieved records to exclude irrelevant studies that did not focus on beer consumption. This process reduced the dataset from 3478 records to a final sample of 2609 articles.

For inclusion, records had to explicitly address beer consumption—either as the main topic or as a key variable examined in relation to relevant correlates or outcomes. We included studies on consumption patterns and trends, studies assessing sociodemographic factors associated with beer consumption, and studies examining associations between beer consumption and health-related outcomes. In line with the WHO definition [19] health was considered broadly as physical, mental, and social well-being, not merely the absence of disease.

Conversely, records that produced false positives because they contained the term “beer” but were unrelated to the beverage were excluded. This included proper nouns (e.g., Beer-Sheva), author surnames, and technical or legal terms (e.g., Beer–Lambert/Beer’s law or the Beers Criteria), as well as spelling artifacts. We also excluded studies focused exclusively on beer-related objects (e.g., containers or glassware), and articles in which beer was only mentioned incidentally and did not represent a substantive variable or meaningful component of the analysis.

### 2.3. Data Processing and Analysis

The bibliometric analysis was performed using the “bibliometrix” package, and its web-based interface, Biblioshiny, implemented in the R (version 4.2.3) statistical environment [16]. These tools enable the systematic mapping of scientific production using quantitative indicators [14,20]. The following indicators were examined: annual publication output; most productive authors; leading institutions and countries; journals with the highest publication volume; keyword co-occurrence patterns; co-authorship and co-citation networks; and the main thematic areas of the field [21].

## 3. Results

This section presents the main bibliometric findings, organized by subheadings. Results are reported concisely, focusing on (i) descriptive indicators of scientific production, (ii) collaboration and intellectual-structure networks, and (iii) thematic patterns and their evolution, along with the key conclusions supported by these analyses.

### 3.1. General Analysis of Publications

**Figure 2:**
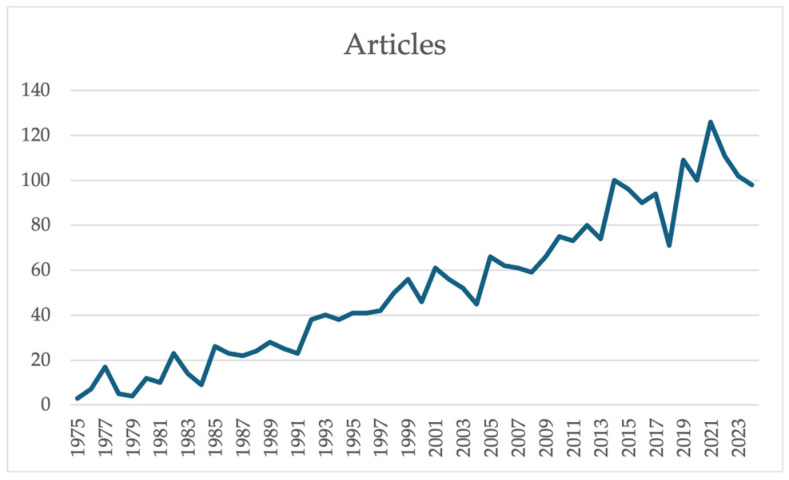
Number of articles published per year between 1974 and 2024.

Annual scientific output on beer consumption showed a sustained upward trend across the study period (Figure 2). Early output (1970s–1990s) was low and irregular. Growth became more marked from 2000 onward, accelerating after 2010, with several years exceeding 70 publications and a peak in 2014.

A marked decline was observed in 2018 relative to adjacent years, followed by a recovery in subsequent years. The highest output of the period occurred in 2021, with 126 indexed documents. Despite year-to-year fluctuations, publication volume increased overall, particularly during the last decade.

### 3.2. Geographic Analysis: Countries with the Highest Scientific Output and International Collaborations

This section examines the geographical distribution of scientific output on beer consumption, considering the total number of publications, the volume of citations received, and the citations per publication ratio (CP), with the aim of assessing both the productivity and scientific impact of the most active countries in this research area.

The highest publication output was observed for the United States, the United Kingdom, Spain, Australia, and Italy. The United States accounted for approximately 30% of all indexed documents and received the largest number of citations (37,508), followed by the United Kingdom (6296), indicating both high research activity and strong international visibility (Table 1).

Regarding the average impact per publication, the Netherlands (48.5), Denmark (48.4), and the United States (47.8) exhibit the highest values, suggesting that, in addition to maintaining sustained research output, their studies tend to receive substantial academic recognition.

**Figure 3:**
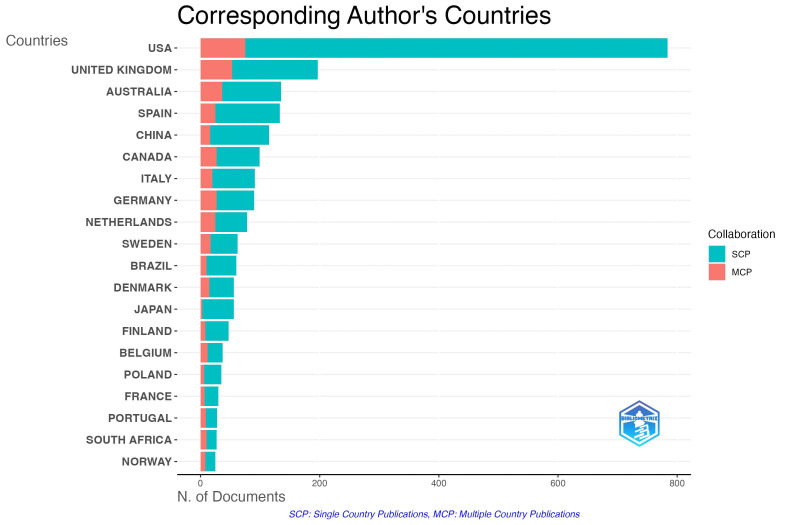
Leading countries in scientific production on beer consumption, distinguishing between single-country publications (SCP) and multiple-country publications (MCP).

Figure 3 extends the country-level analysis by showing publication volume alongside international collaboration, according to the affiliation of the corresponding author. Publications are classified as single-country publications (SCP) when all authors are affiliated with institutions from the same country, and as multiple-country publications (MCP) when at least two countries are represented. The United States again leads in output, with production dominated by SCP. In contrast, the United Kingdom, Australia, and the Netherlands show a more balanced SCP–MCP profile, indicating greater engagement in international collaboration networks.

This pattern reveals differences in national research strategies, highlighting that both publication volume and openness to international collaboration are key factors enhancing the visibility and scientific impact of academic output.

**Figure 4:**
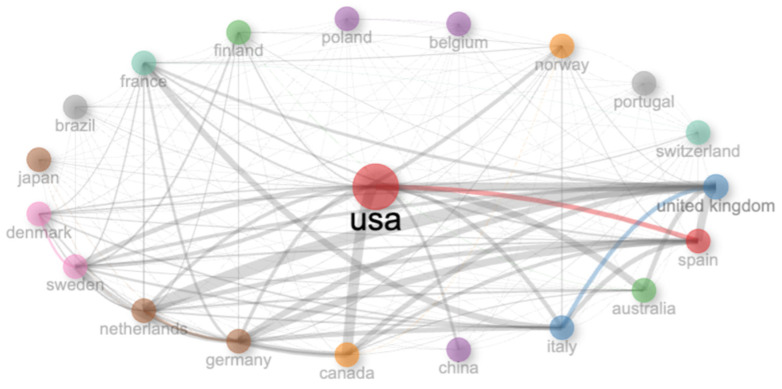
International collaboration network among countries in beer consumption research.

Figure 4 presents the international collaboration network among countries involved in research on beer consumption and health, based on a co-authorship analysis. The size of each node represents the scientific productivity of a country, while the links between nodes indicate collaborative relationships through co-authored publications, with thicker lines reflecting stronger collaboration intensity. The network reveals a highly interconnected global research structure, with the United States occupying a central position as the main hub of international collaboration. This centrality reflects both its high publication output and its extensive collaborative links with multiple countries, particularly with the United Kingdom, Spain, and several European research systems.

European countries such as the United Kingdom, Italy, Germany, the Netherlands, and the Nordic countries also display strong participation in the collaboration network, forming a dense cluster of interactions. From a network perspective, the central position of the United States suggests a key role in facilitating knowledge diffusion and coordinating international research collaborations within the field.

### 3.3. Source Analysis: Productivity and Relevance

#### 3.3.1. Most Productive and Cited Sources

The analysis of sources at the global level reveals that the journals with the highest productivity in the field of beer consumption research are Food Chemistry, Addiction, Alcoholism: Clinical and Experimental Research, Journal of Studies on Alcohol, and Alcohol and Alcoholism (Table 2). These journals account for a substantial volume of publications on the topic, with Food Chemistry standing out prominently, with a total of 287 publications, followed by Addiction, with 106.

However, when examining the most cited journals within the analyzed corpus itself (local citations), a reconfiguration of leadership is observed. In this context, Food Chemistry declines to the eighth position, while Addiction, Alcoholism: Clinical and Experimental Research, and Journal of Studies on Alcohol occupy the top ranks. These journals, which are more directly focused on health sciences, addiction research, and alcohol studies, demonstrate greater thematic proximity to beer consumption, which may explain their higher centrality and influence within the specific research field analyzed.

#### 3.3.2. Additional Related Indicators

Indicators such as the m-index, which adjusts impact according to the time elapsed since the first publication, identify Addiction (1.303) and Nutrients (1.357) as the journals with the highest relative annual impact rates. The latter case is particularly noteworthy, as Nutrients began publishing on this topic in 2012, suggesting a rapid growth in its influence within the field (Table 3).

Additionally, journal influence was assessed using the h-index and g-index, which further highlighted the leading outlets. Food Chemistry ranked first on both metrics (h = 57; g = 85), indicating not only high productivity but also consistent citation impact over time. In contrast, local citation analysis (citations within the study corpus) emphasized journals more directly aligned with alcohol and addiction research—such as Addiction, Alcoholism: Clinical and Experimental Research, and the Journal of Studies on Alcohol—which received comparatively more within-field recognition than more generalist journals.

**Figure 5:**
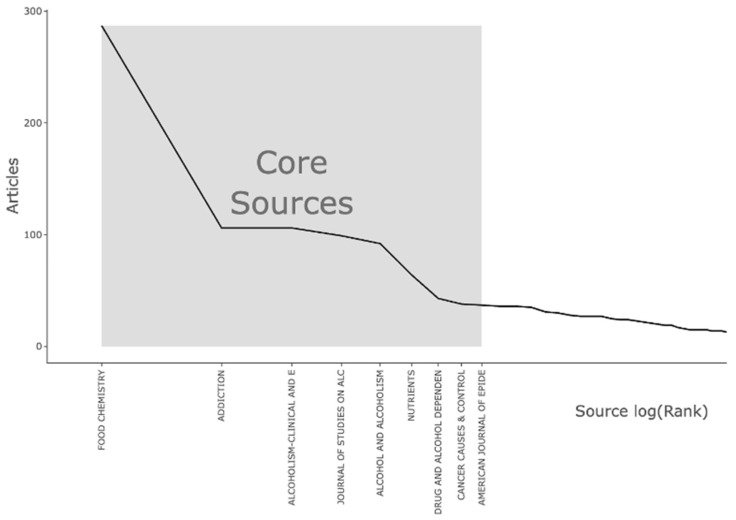
Bradford’s Law.

Finally, source dispersion was examined using Bradford’s Law, revealing a typical distribution pattern. A core group of only nine journals accounted for approximately one-third of the total articles, followed by a second zone comprising 53 sources and a third zone including 454 sources, which collectively contributed similar volumes of publications. The journals forming this Bradford core include *Food* *Chemistry*, *Addiction*, *Alcoholism: Clinical and Experimental Research*, *Journal of Studies on Alcohol and Drugs*, *Alcohol and Alcoholism*, *Nutrients*, *Drug and Alcohol Dependence*, *Cancer Causes & Control*, and *American Journal of Epidemiology*. As illustrated in Figure 5, the steep decline in article frequency after the core journals indicates that the remaining publications are widely dispersed across a large number of journals, each contributing relatively few articles. This pattern suggests that research on beer consumption and health is concentrated in a limited number of specialized journals while simultaneously extending across a broad and fragmented peripheral literature.

### 3.4. Institutional Analysis: Leading Institutions and Scientific Output

**Figure 6:**
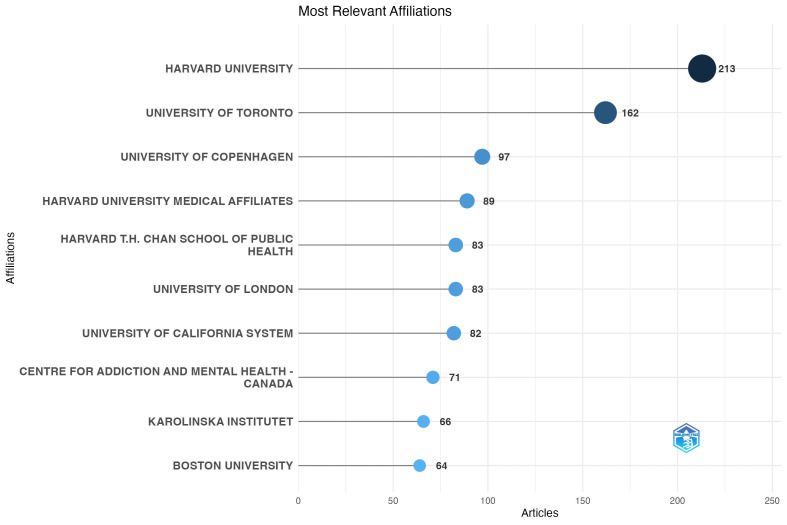
Number of publications by the most relevant institutions.

The institutional analysis indicates that Harvard University leads scientific production on beer consumption, with 213 publications, followed by the University of Toronto (162) and the University of Copenhagen (97). The presence of other entities affiliated with Harvard, such as Harvard Medical Affiliates and the Harvard T.H. Chan School of Public Health, is also noteworthy, reinforcing its central role in this research area, particularly from a public health perspective (Figure 6).

### 3.5. Author Analysis: Leading Authors and Scientific Output

Authorship analysis makes it possible to identify the researchers with the strongest presence in scientific production on beer consumption, as well as to evaluate the impact of their contributions and their temporal evolution. Among the most productive authors are Kerr W.C. (34 publications), Rehm J. (27), and Greenfield T.K. (22), followed by Room R., Shield K.D., Anderson P., and other scholars with well-established research trajectories in the field of alcohol and public health.

**Figure 7:**
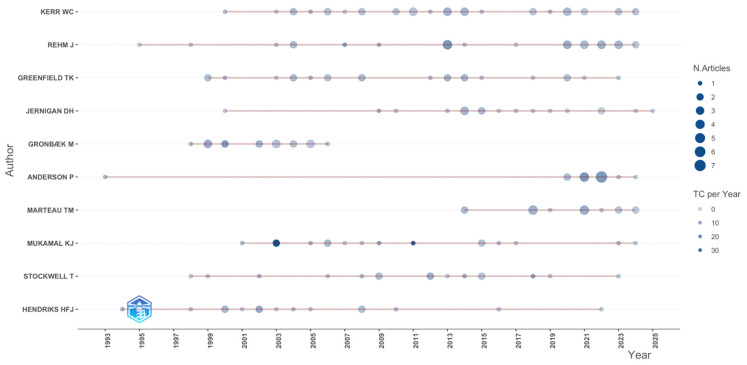
Author scientific production over time (years) in beer consumption research.

The graphical representation of scientific output over time (Author Production Over Time) provides insight into the evolution of these authors’ research activity (Figure 7). For instance, Kerr W.C. has maintained sustained productivity from the early 2000s to the present, with particularly notable activity during the past decade. Rehm J. and Greenfield T.K. also demonstrate consistent scholarly presence, especially since the mid-2000s, with peaks in productivity during key periods. In contrast, authors such as Grønbæk M. and Mukamal K.J. concentrated their research output within more limited timeframes, particularly between 2000 and 2010, whereas others, including Anderson P. and Marteau T.M., have shown more prominent activity in recent years.

In terms of impact, a clear alignment between productivity and citation frequency can be observed. Three of the most highly cited authors—Rehm J., Kerr W.C., and Greenfield T.K.—also rank among the most prolific, suggesting not only substantial quantitative output but also significant academic recognition and influence within the scientific community. This analysis highlights the existence of well-established and active research clusters focused on beer consumption and identifies key opinion leaders whose work shapes research trends and exerts considerable influence within the field.

### 3.6. Document Analysis

Table 4 presents the ten most globally cited articles, thereby illustrating the impact and influence of specific works within the analyzed research field.

Within the selected sample, the article by Rimm [22], entitled “Moderate alcohol intake and lower risk of coronary heart disease: meta-analysis of effects on lipids and hemostatic factors”, published in BMJ, ranks first with a total of 1002 citations, demonstrating its high visibility and substantial impact within the scientific community.

The indicator “Total Citations per Year” provides additional insight into the sustained relevance of individual publications over time. In this regard, the article by Wagenaar [23], entitled “Effects of beverage alcohol price and tax levels on drinking: a meta-analysis of 1003 estimates from 112 studies”, published in Addiction, stands out with an average of 39.82 citations per year, indicating a particularly strong and consistent influence since its publication.

Furthermore, the “Normalized Total Citations” metric enables comparison of the relative impact of documents by adjusting citation counts according to publication year and other contextual factors. In this case, the article by Salvini et al. [24] entitled “Food-Based Validation of a Dietary Questionnaire: The Effects of Week-to-Week Variation in Food Consumption”, presents the highest normalized citation score (16.15), indicating a particularly strong influence relative to its scientific context at the time of publication.

Taken together, these three indicators—total citations, average annual citations, and normalized citations—provide a complementary and more comprehensive assessment of document impact. This approach makes it possible to identify not only the most highly cited articles in absolute terms but also those with particularly significant relative influence within their respective scientific contexts.

The analysis of the most cited documents within the thematic corpus reveals a combination of studies with high global impact and others exerting more field-specific influence in beer consumption research (Table 5). Once again, the article by Wagenaar et al. [23] published in Addiction, ranks first with 73 local citations, indicating a strong presence and influence within the analyzed domain, although its local-to-global citation ratio (LC/GC Ratio) remains moderate (10.78%).

In contrast, the study by Smart [25] exhibits the highest LC/GC Ratio (59.02%), suggesting that, although its global impact is relatively limited, it has been particularly influential within this specific research field.

Finally, the article by Arranz [10], published in Nutrients, presents one of the highest normalized local citation values (22.5). This finding, together with its relatively recent publication date, points to a document of increasing relevance in recent years, consistent with the observed upward trend in publication output in this journal, as reflected in the longitudinal analysis of source productivity.

### 3.7. Thematic Analysis

#### 3.7.1. Most Frequent Keywords

Although keyword metadata were rated as “poor” in the initial Biblioshiny data-quality assessment, keyword analysis was retained to provide an exploratory overview of the field’s thematic structure. Both Author Keywords and Keywords Plus were examined. However, no manual recoding or substantive terminological harmonization of keyword fields was performed prior to the keyword co-occurrence and thematic analyses. Therefore, keyword-based results should be interpreted with caution.

The most frequent terms in the All Keywords category were alcohol (592 occurrences), consumption (455), beer (401), drinking (381), risk (320), wine (204), mortality (200), health (161), and alcohol consumption (136). These terms reflect the multidisciplinary nature of the field, encompassing public health perspectives, consumption behaviors, and risks associated with alcoholic beverage intake.

#### 3.7.2. Keyword Co-Occurrence Network

**Figure 8:**
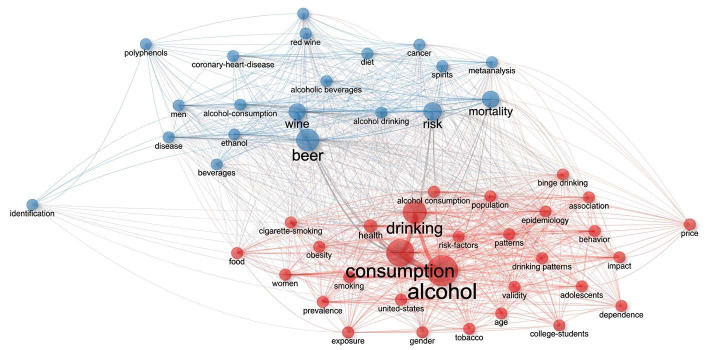
Keyword co-occurrence network (All Keywords).

The keyword co-occurrence network enables the identification of the principal thematic domains within the literature on beer consumption. The network visualization reveals two distinct clusters, represented in red and blue, reflecting thematic groupings based on the frequency and co-occurrence patterns of keywords (Figure 8).

The red cluster, which is denser, includes keywords such as alcohol, consumption, drinking, health, risk factors, adolescents, and behavior. This cluster suggests a thematic focus centered on consumption patterns and their public health implications, with particular emphasis on sociodemographic variables (e.g., age, gender, university students) and behavioral factors (e.g., dependence, smoking, obesity). This cluster appears to be primarily oriented toward the epidemiological and behavioral analysis of alcohol consumption and its associated risks.

In contrast, the blue cluster is more closely associated with biomedical and nutritional dimensions. It includes terms such as beer, wine, polyphenols, mortality, coronary heart disease, diet, and cancer, indicating a research focus on the health effects of alcoholic beverage consumption—particularly beer—on cardiovascular health and chronic disease risk. The presence of terms such as meta-analysis further reflects an emphasis on evidence synthesis within this thematic domain.

In terms of centrality and frequency, the keywords alcohol, consumption, and drinking emerge as the most prominent nodes, serving as structural connectors between the two clusters. The connectivity among terms also highlights the presence of interdisciplinary research linking individual behavioral factors with biomedical and social determinants of alcohol consumption.

Overall, this analysis demonstrates the existence of at least two dominant research perspectives: a clinical–nutritional approach and a population-based behavioral approach, both of which exhibit important areas of intersection that may inform future integrative research.

#### 3.7.3. Thematic Analysis Using Clustering Techniques

**Figure 9:**
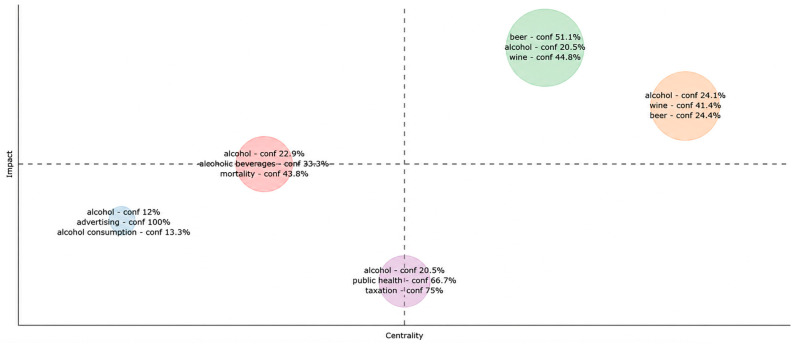
Thematic cluster in alcohol consumption research based on centrality and density.

To identify the main thematic research areas, a keyword coupling analysis was conducted and visualized through a thematic map (Figure 9), in which themes are evaluated based on their centrality (structural importance within the field) and density (level of thematic development).

The most prominent cluster, located in the upper-right quadrant, includes terms such as beer, alcohol, and wine, indicating that comparative studies of alcoholic beverages represent a central and well-established research theme. Another cluster of considerable interest comprises terms such as public health, alcohol, and taxation. Although this cluster shows lower density, its high centrality suggests that it represents an emerging research area with strong potential for future development.

In contrast, other clusters appear less integrated into the core structure of the field, including those focused on advertising and on the relationship between alcoholic beverages and mortality. These themes may reflect more specialized research areas or topics that have experienced comparatively lower recent development.

## 4. Discussion

This study aimed to provide a comprehensive overview of the scientific literature examining beer consumption in relation to health over a 50-year period. The findings show a sustained increase in scientific output, particularly from the 2000s onward and reveal a multidisciplinary field shaped by public health, epidemiology, nutrition, addiction research, food science, and behavioral sciences. This growth can be interpreted as reflecting increasing scientific interest in the determinants and consequences of alcohol consumption from a public health perspective, as well as a progressive broadening of the research focus: from biomedical and cardiovascular effects toward wider dimensions related to drinking patterns, social inequalities, mental health, taxation, and preventive policies [4,5,7].

Several bibliometric studies have mapped alcohol research more broadly, reporting sustained growth in publications, a concentration of output in high-income countries, and strong contributions from established alcohol epidemiology networks. The present beer-focused map is consistent with these patterns—including the increase in output since the 2000s and the predominance of the United States and other high-income countries—but it also highlights a distinctive product-oriented strand (e.g., food chemistry and brewing-related outlets) that is less prominent in general alcohol bibliometrics. The observed thematic transition—from earlier cardiovascular/biomedical and composition-related topics toward drinking patterns, public health, taxation, and prevention—also mirrors wider shifts in alcohol and addiction research toward population-level risk and policy responses [4,5,11,13,26,27]. Within the nutrition-epidemiology strand, the prominence of classic cardiovascular papers and the subsequent rise of methodological critiques of putative protective effects is aligned with the evolving evidence base challenging J-shaped interpretations [11,12,28,29].

In this context, beer occupies a particularly relevant position. As one of the most widely consumed alcoholic beverages worldwide, its study cannot be limited to its nutritional or biochemical properties but should also be situated within the social and cultural patterns of alcohol consumption. Its high availability, social acceptability, and frequent presence in leisure, sport, socialization, and everyday life contexts make beer a common and socially normalized exposure [9,30]. Therefore, the literature on beer and health simultaneously reflects questions related to nutrition, alcohol epidemiology, drinking behavior, and public health. Beer-related research is dispersed across food science, nutrition, epidemiology, addiction research, behavioral sciences, and public health; mapping this literature separately helps clarify how beer consumption has been studied as a health-related phenomenon rather than only as a food product or as an undifferentiated component of total alcohol intake.

The increase in publications observed in recent years may also be related to growing concern about alcohol-related harm and the consolidation of evidence-based prevention strategies. It is noteworthy that scientific output appears to have maintained an upward trend even during periods of major global disruption, such as the COVID-19 pandemic. Although the present study does not specifically analyze the impact of the pandemic on research on beer and health, the continuity of scientific output during this period suggests that alcohol remained a relevant priority within the public health and behavioral research agenda.

The geographical distribution of scientific production shows a clear predominance of high-income countries, especially the United States, the United Kingdom, Spain, Australia, Italy, Canada, the Netherlands, Germany, Denmark, and China. In particular, the United States stands out as the most productive country and the one with the highest citation volume, which may be explained by its strong scientific infrastructure, well-established funding systems, large epidemiological cohorts, and highly internationalized academic networks. This pattern is consistent with the concentration of biomedical and public health research in countries with high scientific and editorial capacity. However, this geographical concentration should also be interpreted critically. The predominance of high-income countries may reflect not only differences in scientific productivity but also broader geopolitical and structural factors, including unequal research funding, stronger academic infrastructures, greater access to large cohort studies, English-language publication practices, and higher visibility in international citation databases [31]. In the field of alcohol and public health research, these asymmetries are particularly relevant because drinking patterns, regulatory frameworks, commercial environments, and alcohol-attributable harms may vary substantially across regions.

Nevertheless, scientific production should not be interpreted only in terms of volume. The results also show differences in relative impact and international collaboration patterns. Some countries with a lower absolute number of publications, such as the Netherlands and Denmark, show a high number of citations per publication, suggesting a more concentrated but highly influential scientific output. International collaboration also appears to be a key element in the configuration of the field. Although the United States occupies a central position in the collaboration network, a considerable proportion of its output remains domestically produced. In contrast, European countries such as the United Kingdom, the Netherlands, Germany, Italy, and the Nordic countries show a relevant presence in international collaboration networks. This finding is consistent with bibliometric literature indicating that international collaboration can increase the visibility, impact, and circulation of scientific knowledge [32,33].

The institutional analysis reinforces this interpretation. The presence of universities and research centers such as Harvard University, the University of Toronto, and the University of Copenhagen suggests that the field is partly structured around institutions with a consolidated trajectory in epidemiology, nutrition, public health, and alcohol research. The relevance of Harvard-affiliated institutions, for example, is consistent with the presence of influential studies on alcohol, drinking patterns, and cardiovascular disease, including classic cohort-based epidemiological research. Similarly, the presence of European and Nordic centers reflects the importance of population-based and public health research traditions that have contributed substantially to the study of alcohol and its consequences.

At the source level, the results show a particularly relevant dual structure. On the one hand, journals such as *Food* *Chemistry* stand out in terms of productivity, reflecting the importance of food science, beer composition, bioactive compounds, and the nutritional or technological aspects of the beverage. On the other hand, the sources with the highest local influence within the corpus—such as *Addiction*, *Alcoholism: Clinical and Experimental Research*, and the *Journal of Studies on Alcohol and Drugs*—belong more clearly to the fields of alcohol research, addiction, epidemiology, and public health. This difference between global productivity and local influence is important because it shows that research on beer and health is not organized exclusively around beer as a food product, but also around alcohol as a health-related and behavioral exposure.

This pattern is also reflected in the most cited documents. Studies by Rimm et al. [22], Mukamal et al. [34], Grønbæk et al. [35], and other cardiovascular studies reflect the historical influence of the hypothesis that low or moderate alcohol consumption may be associated with a lower risk of coronary heart disease. This line of research has often been linked to the debate on the J-shaped relationship between alcohol and health, as well as to possible biological mechanisms related to lipid profiles, hemostatic factors, or compounds present in specific alcoholic beverages. In the case of beer, this tradition has been connected to interest in polyphenols, dietary context, and differences between beer, wine, and spirits [8,10,36].

However, the influence of these documents should be interpreted with caution. The bibliometric prominence of studies on cardiovascular protection does not necessarily imply causal consensus. In recent decades, the interpretation of the potential beneficial effects of moderate alcohol consumption has been subject to increasing methodological debate. Recent studies have questioned whether the protective associations observed in some studies reflect a causal effect of alcohol or, rather, biases derived from residual confounding, reverse causation, reference-group selection, or the inappropriate classification of former drinkers as abstainers [11,12]. Therefore, the most cited documents should be understood not only as influential evidence, but also as points of condensation of scientific debates that have evolved over time.

One of the main contributions of the present study is to show that the literature on beer and health does not constitute a homogeneous field. Consistent with the journals’ scope and common research topics, the field appears to be organized around three dominant but partially overlapping perspectives: (i) beer-specific nutritional and biochemical research examines beer as a distinct beverage, focusing on its composition, polyphenol content, fermentation-derived compounds, food matrix, non-alcoholic alternatives, and potential beverage-specific mechanisms; (ii) a comparative epidemiology of alcoholic beverages compares beer with wine, spirits, or total alcohol intake in relation to health outcomes, drinking patterns, and population subgroups, although such comparisons may also reflect differences in diet, socioeconomic position, cultural context, drinking frequency, and drinking occasion; and (iii) general alcohol and public health research examines ethanol exposure, total alcohol intake, heavy episodic drinking, alcohol-attributable burden, prevention policies, and population-level harm, with beer often included as one beverage category.

Within and across these perspectives, however, a further interpretative distinction is needed between beer-specific evidence, beverage-comparative evidence, and general alcohol or ethanol-related evidence. This distinction matters because associations between beer consumption and health should not automatically be attributed to beer-specific properties. They may reflect ethanol exposure, drinking patterns, social determinants, dietary patterns, socioeconomic position, or drinking context. Conversely, potentially beer-specific mechanisms related to beer composition, fermentation-derived compounds, food matrices, or non-alcoholic alternatives cannot be inferred from studies examining total alcohol intake alone. Therefore, conclusions should be framed according to the type of evidence being analyzed: beer-specific interpretation when beer is studied as a distinct product, beverage-comparative interpretation when beer is compared with other alcoholic drinks, and alcohol-epidemiological interpretation when beer is analyzed as part of total alcohol intake.

The thematic structure observed in the keyword co-occurrence analysis supports this interpretation. A first cluster reflects a clinical–nutritional perspective, with terms related to beer, wine, polyphenols, coronary heart disease, diet, cancer, and mortality. This line of research is consistent with previous studies examining the potential cardiometabolic effects of moderate beer consumption and the possible role of polyphenols and other beverage-specific compounds [8,10,36]. A second cluster reflects a socio-epidemiological and behavioral perspective, with terms such as alcohol, consumption, drinking, risk, adolescents, behavior, public health, and taxation. This perspective is aligned with broader alcohol research, where drinking patterns, risk behaviors, prevention strategies, social determinants, and policy measures are central concerns [4,5,7]. The coexistence of these clusters illustrates a central tension in the field: beer is simultaneously studied as a fermented beverage with specific nutritional components and as a widely consumed alcoholic product contributing to alcohol-related harm.

The interpretation of beer-related research should also be situated within the classic methodological challenges of alcohol epidemiology. Much of the literature on alcohol and health is observational and therefore vulnerable to residual confounding, reverse causation, and misclassification of drinking status. These issues are especially relevant in the literature on moderate consumption, cardiovascular disease, and all-cause mortality. One of the most widely discussed sources of bias is the so-called “sick-quitter” effect, which occurs when former drinkers who stopped consuming alcohol because of health problems are included in the abstainer or non-drinker reference group [37]. If these former drinkers have poorer baseline health, comparisons may artificially create an apparent advantage for moderate drinkers over abstainers. Recent meta-analytic evidence indicates that apparent benefits of low-volume drinking are more likely to appear in studies with a greater risk of reference-group misclassification and other design limitations, whereas higher-quality studies show weaker or null protective associations [28,29].

Measurement error is also a central issue. Alcohol consumption is usually assessed through self-report, which introduces possible recall bias, social desirability bias, underreporting, and temporal instability. In addition, average-volume measures may obscure clinically relevant differences between drinking patterns. Consuming a given weekly amount of alcohol distributed across several meals is not equivalent to consuming the same amount during one or two heavy episodic drinking occasions [38]. This issue is particularly relevant for beer, which is frequently consumed in social, recreational, or group contexts where drinking patterns, settings, and co-occurring behaviors may substantially influence health risk.

These methodological debates help interpret another important shift in the field: the move from an interpretation centered on possible cardioprotective effects of moderate drinking toward a more cautious perspective focused on population-level risk. Global Burden of Disease analyses have played a central role in this shift. The GBD 2016 study concluded that alcohol use was a major risk factor for deaths and disability-adjusted life-years worldwide and argued that the level of consumption minimizing overall health loss was zero [26]. Subsequently, the GBD 2020 analysis refined this conclusion by showing that risk varies by age, sex, geographical region, and disease profile, with a particularly high burden among young adults [13]. This evolution does not invalidate beer-specific research, but it does require caution when interpreting findings on moderate consumption, polyphenols, or cardiometabolic outcomes, placing them within the broader evidence on ethanol, drinking patterns, vulnerable populations, and alcohol-attributable harm.

The prominence of public health, taxation, and preventive policy within the corpus also deserves attention. The high local impact of Wagenaar et al.’s meta-analysis on alcohol prices and taxes shows that policy-oriented research constitutes a central component of the literature on beer, alcohol, and health [23]. Alcohol taxation, minimum pricing, marketing restrictions, availability regulation, drink-driving countermeasures, and health warnings have been widely discussed as effective strategies to reduce alcohol-related harm [4,27]. Within this framework, beer is especially relevant because of its high prevalence of consumption, cultural acceptability, wide availability, and frequent perception as a lower-risk beverage.

The interpretation of beer-related literature should also consider commercial determinants of health. Beer consumption is shaped not only by individual preferences or cultural practices, but also by pricing strategies, advertising, sponsorship, packaging, brand positioning, product innovation, and market dynamics. The presence of beer in leisure, sport, socialization, and everyday contexts may reinforce its normalization and reduce risk perception. From this perspective, alcohol-related harm cannot be addressed only through individual education or consumption recommendations but requires attention to the commercial and regulatory environments in which drinking behaviors occur [39].

Likewise, because alcohol-related research may intersect with commercial interests, transparency regarding funding and conflicts of interest is essential. This is especially important in beverage-specific research, where interpretations regarding potential benefits of moderate consumption, product differentiation, non-alcoholic alternatives, or beverage-specific effects may have commercial relevance. Transparency does not invalidate scientific findings, but it is necessary to assess the independence, credibility, and public health interpretation of the available evidence. Previous research has highlighted the importance of critically examining alcohol industry involvement in both science and policymaking, especially because industry interests may not always align with population-level harm-reduction goals [40]. Therefore, future literature reviews and meta-analyses should assess whether the content, interpretation, and conclusions of the available studies differ according to the declared conflicts of interest and funding sources. This would help determine whether funders may influence the research process and the conclusions drawn in this field.

The findings of this study have several practical implications. For researchers, the bibliometric map helps identify the main intellectual traditions, influential journals, leading authors, productive institutions, and emerging topics in the field. This may facilitate more focused literature reviews, promote collaboration, and clarify how research on beer is distributed across nutrition, epidemiology, addiction science, behavioral sciences, and public health. For public health professionals, the results emphasize the importance of interpreting beer consumption not only in terms of potential beverage-specific properties but also in relation to drinking patterns, population vulnerability, commercial determinants, and alcohol-related harm. In this regard, greater attention should be paid to vulnerable and underrepresented populations, including adolescents, university students, older adults, women, people with mental health problems, and populations outside high-income Western countries. In addition, differentiating between alcoholic beer, non-alcoholic beer, low-alcohol beer, craft beer, and industrial beer may be relevant for public health interpretation, as these products may differ in composition, consumption context, commercial positioning, and potential implications for prevention. For policymakers, the prominence of taxation and public policy indicates that evidence on beer should be integrated into broader alcohol harm-reduction strategies, rather than treated separately from the general alcohol policy agenda.

Future research should better differentiate between alcoholic beer, non-alcoholic beer, low-alcohol beer, craft beer, and industrial beer, as these products may differ in composition, consumption context, commercial positioning, and public health implications. Greater attention should also be paid to vulnerable and underrepresented populations, including adolescents, university students, older adults, women, people with mental health problems, and populations outside high-income Western countries.

The analysis also identifies several research gaps. First, more studies are needed that clearly distinguish beer-specific effects from ethanol-related effects and from lifestyle- or diet-related confounding. Second, future research should better differentiate between alcoholic beer, non-alcoholic beer, low-alcohol beer, craft beer, and industrial beer, as these products may differ in composition, consumption context, commercial positioning, and public health implications [41]. Third, longitudinal and methodologically robust studies are needed to clarify whether beer consumption influences later health outcomes or whether pre-existing health conditions shape drinking behavior, as well as to address abstainer misclassification, residual confounding, and drinking-pattern heterogeneity. Fourth, greater attention should be paid to vulnerable and underrepresented populations, including adolescents, university students, older adults, women, people with mental health problems, and populations outside high-income Western countries. Research on university students, for example, shows that alcohol consumption is closely linked to social context and institutional environments, reinforcing the need for population-specific approaches [42].

Finally, several limitations should be acknowledged. First, the analysis was restricted to English-language publications indexed in the Web of Science Core Collection. WoS provides high-quality citation metadata and strong compatibility with bibliometric tools such as Bibliometrix, which supports reproducibility and analytical consistency [14,16]. However, the use of a single database may introduce coverage bias, especially in a multidisciplinary field spanning nutrition, food science, psychology, epidemiology, addiction research, medicine, nursing, and public health. Relevant studies indexed only in Scopus, PubMed/MEDLINE, Embase, PsycINFO, or regional databases may not have been included. Second, although screening was conducted to ensure that included documents addressed beer consumption as a relevant variable, the boundaries between beer-specific research and general alcohol research are not always clear. This heterogeneity is not only a limitation but also an important finding, as it reflects the hybrid nature of the field. Third, keyword metadata were classified as suboptimal during the data-quality assessment performed in Biblioshiny. Since no manual recoding or substantive harmonization of keyword fields was conducted, inconsistencies in Author Keywords and Keywords Plus may have affected the precision of co-occurrence and thematic clustering analyses. Therefore, thematic results based on keyword metadata should be interpreted with caution. Lastly, bibliometric analysis can identify patterns of productivity, influence, collaboration, and thematic prominence, but it does not directly assess the methodological quality, causal validity, or risk of bias of individual studies [14,20]. Consequently, the findings of this study do not allow causal conclusions to be drawn regarding the effects of beer consumption on health. Rather, they should be interpreted as a scientific map of the field and not as a direct synthesis of evidence on the health consequences of beer consumption.

## 5. Conclusions

This bibliometric study provides an integrated overview of the scientific landscape on beer consumption and health over the last five decades. The findings show sustained growth in scientific output, a strong concentration of research in high-income countries, and the coexistence of nutritional, epidemiological, behavioral, and public health perspectives.

Overall, the results suggest that beer-related health research is a multidisciplinary and heterogeneous field, situated between beverage-specific research and broader alcohol epidemiology. This distinction is important for interpreting the evidence and for guiding future studies. Further research should adopt more robust methodological designs, clearly differentiate beer-specific effects from general alcohol-related effects, and pay greater attention to drinking patterns, vulnerable populations, non-alcoholic and low-alcohol alternatives, and policy-relevant public health questions.

## Figures and Tables

**Table 1:** List of countries, total citations, and citations per publication.

Country	Total Citations	Citations per Publication (CP)	Total Publications
United States	37,508	47.8	2010
United Kingdom	6296	32.0	549
Spain	4456	33.5	482
Australia	3790	28.1	391
Netherlands	3782	48.5	244
Canada	3484	35.2	340
Germany	3095	34.4	303
Italy	3067	33.7	349
Denmark	2711	48.4	207
China	2389	20.8	343

**Table 2:** Leading journals in beer consumption research: publication output and local citation impact.

Most Relevant Sources		Most Local Cited Sources	
Journal	Article Count	Journal	Article Count
Food Chemistry	287	Addiction	2349
Addiction	106	Alcoholism: clinical and experimental research	1935
Alcoholism: clinical and experimental research	106	Journal studies on alcohol	1704
Journal studies on alcohol	99	Journal of Agricultural and Food Chemistry	1578
Alcohol and alcoholism	92	American Journal of Epidemiology	1377
Nutrients	64	Lancet	1369
Drug and alcohol dependence	43	The American Journal of Clinical Nutrition	1201
Cancer causes & control	38	Food Chemistry	1134
American Journal of Epidemiology	37	Alcohol and alcoholism	1053

**Table 3:** Bibliometric indicators of the leading sources on beer consumption.

Source	h-Index	g-Index	m-Index	Total Citations (TC)	Number of Publications (NP)	Publication Start Year (PY_Start)
Food Chemistry	57	85	1.163	10,913	287	1977
Addiction	43	75	1.303	5963	106	1993
Alcoholism: Clinical and Experimental Research	38	59	1.086	4171	106	1991
Journal of Studies on Alcohol	36	54	0.706	3478	99	1975
American Journal of Epidemiology	31	37	0.721	2803	37	1983
Alcohol and Alcoholism	29	50	0.725	2807	92	1986
Cancer Causes & Control	25	38	0.694	1603	38	1990
American Journal of Clinical Nutrition	23	30	0.469	2384	30	1977
European Journal of Clinical Nutrition	22	36	0.647	1464	36	1992
Nutrients	19	33	1.357	1209	64	2012

**Table 4:** Most globally cited documents.

Paper	Total Citations	TC per Year	Normalized TC	DOI
RIMM EB, 1999, BMJ-BRIT MED J	1002	37.11	12.83	10.1136/bmj.319.7224.1523
SALVINI S, 1989, INT J EPIDEMIOL	917	24.78	16.15	10.1093/ije/18.4.858
RIMM EB, 1996, BRIT MED J	778	25.93	12.97	10.1136/bmj.319.7224.1523
STAMPFER MJ, 1988, NEW ENGL J MED	726	19.11	12.02	10.1056/NEJM198808043190503
WAGENAAR AC, 2009, ADDICTION	677	39.82	13.31	10.1111/j.1360-0443.2008.02438.x
MUKAMAL KJ, 2003, NEW ENGL J MED	589	25.61	8.77	10.1056/NEJMoa022095
BRIEN SE, 2011, BMJ-BRIT MED J	558	37.2	13.23	10.1136/bmj.d636
JACKA FN, 2010, AM J PSYCHIAT	538	33.63	11.54	10.1176/appi.ajp.2009.09060881
YANO K, 1977, NEW ENGL J MED	525	10.71	10.11	10.1056/NEJM197708252970801
GRONBAEK M, 1995, BRIT MED J	503	16.23	9.21	10.1136/bmj.310.6988.1165

**Table 5:** Documents with the highest local impact.

Document	Global Citations	Local Citations	LC/GC Ratio (%)	Normalized Local Citations	Normalized Global Citations	DOI
WAGENAAR AC, 2009, ADDICTION	677	73	10.78	21.41	13.31	10.1111/j.1360-0443.2008.02438.x
KLATSKY AL, 1990, BRIT J ADDICT	152	51	33.55	11.09	3.56	10.1111/j.1360-0443.1990.tb01604.x
GRONBAEK M, 1995, BRIT MED J	503	51	10.14	9.33	9.21	10.1136/bmj.310.6988.1165
RIMM EB, 1999, BMJ-BRIT MED J	1002	48	4.79	8.96	12.83	10.1136/bmj.319.7224.1523
TJONNELAND A, 1999, AM J CLIN NUTR	227	46	20.26	8.59	2.91	10.1093/ajcn/69.1.49
ARRANZ S, 2012, NUTRIENTS	368	45	12.23	22.5	9.85	10.3390/nu4070759
GRONBÆK M, 2000, ANN INTERN MED	385	41	10.65	7.43	6.34	10.7326/0003-4819-133-6-200009190-00008
MUKAMAL KJ, 2003, NEW ENGL J MED	589	38	6.45	8.37	8.77	10.1056/NEJMoa022095
DE GAETANO G, 2016, NUTR METAB CARDIOVAS	189	38	20.11	21.92	7.62	10.1016/j.numecd.2016.03.007
SMART RG, 1996, J STUD ALCOHOL	61	36	59.02	13.42	1.02	10.15288/jsa.1996.57.77

## Data Availability

Not applicable.
